# Deployment of precise and robust sensors on board ISS—for scientific experiments and for operation of the station

**DOI:** 10.1007/s00216-016-9789-0

**Published:** 2016-08-15

**Authors:** Christian Stenzel

**Affiliations:** Airbus DS GmbH—TSPOE, Claude-Dornier-Str. 1, 88090 Immenstaad, Germany

**Keywords:** Sensors, Coupling of sensors, Measurement chain, Research under micro-gravity, Sensors for life sciences, Oxygen sensors

## Abstract

The International Space Station (ISS) is the largest technical vehicle ever built by mankind. It provides a living area for six astronauts and also represents a laboratory in which scientific experiments are conducted in an extraordinary environment. The deployed sensor technology contributes significantly to the operational and scientific success of the station. The sensors on board the ISS can be thereby classified into two categories which differ significantly in their key features: (1) sensors related to crew and station health, and (2) sensors to provide specific measurements in research facilities. The operation of the station requires robust, long-term stable and reliable sensors, since they assure the survival of the astronauts and the intactness of the station. Recently, a wireless sensor network for measuring environmental parameters like temperature, pressure, and humidity was established and its function could be successfully verified over several months. Such a network enhances the operational reliability and stability for monitoring these critical parameters compared to single sensors. The sensors which are implemented into the research facilities have to fulfil other objectives. The high performance of the scientific experiments that are conducted in different research facilities on-board demands the perfect embedding of the sensor in the respective instrumental setup which forms the complete measurement chain. It is shown that the performance of the single sensor alone does not determine the success of the measurement task; moreover, the synergy between different sensors and actuators as well as appropriate sample taking, followed by an appropriate sample preparation play an essential role. The application in a space environment adds additional challenges to the sensor technology, for example the necessity for miniaturisation, automation, reliability, and long-term operation. An alternative is the repetitive calibration of the sensors. This approach, however, increases the operational overhead significantly. But meeting especially these requirements offers unique opportunities for testing these sensor technologies in harsh and dedicated environments which are not available on Earth, therefore pushing the related technologies and methodologies to their limits. The scientific objectives for selected experiments, representing a wide range of research fields, are presented, including the instrument setups and the implemented sensor technologies, and where available, the first scientific results are presented.

## Introduction

This article is concerned with the deployed sensor technology on board the International Space Station ISS, as used for the operation of the station and for monitoring and control scientific experiments in various research fields which are performed both inside and outside the space station. In Chapter 2, the ISS is introduced, and its main features and major operational parameters are described. Chapter 3 gives a brief overview on the main objectives for conducting scientific experiments under the special conditions of reduced gravity and the corresponding research fields. In Chapter 4, modern sensor technologies are categorised more generally with respect to their interplay with the complete measurement chain. In Chapter 5, eight selected sensor systems are described in more detail with respect to their design, function, and implementation into the respective experimental setup. The first results of these experiments are reported where available. A critical review is presented in Chapter 6 addressing basic commonalities, a comparison to terrestrial systems, and an outlook on potential spin-off into scientific facilities on ground and even into commercial devices.

## International Space Station

The impetus for building the International Space Station (ISS) was created by the foreign ministers of the major countries in the UNO Security Council in the late 1990s. This internationally operated space station should represent the largest man-made technical device for civil utilisation and should indicate a clear symbolic act on the level of technical enterprises to end the cold war between the super powers. Astronautics and space flight had been prominent fields for technical and military competition during the cold war. In the meantime, both Russian and the American lunar programs have to be regarded from a historical point of view as a military competition rather than as an endeavour driven by scientific research objectives [1, 2]. Hence, the two major players in this context, the United States of America and the Russian Republic with their vast experience in space transportation and engineering, adopted this large programme with the aim to build the ISS as a show of international cooperation instead of enduring confrontation in the space sector. Japan, Canada, and the European Space Agency joined this collaboration later, leading to the participation of the 26 countries which contribute actively to the ISS.

**Fig. 1:**
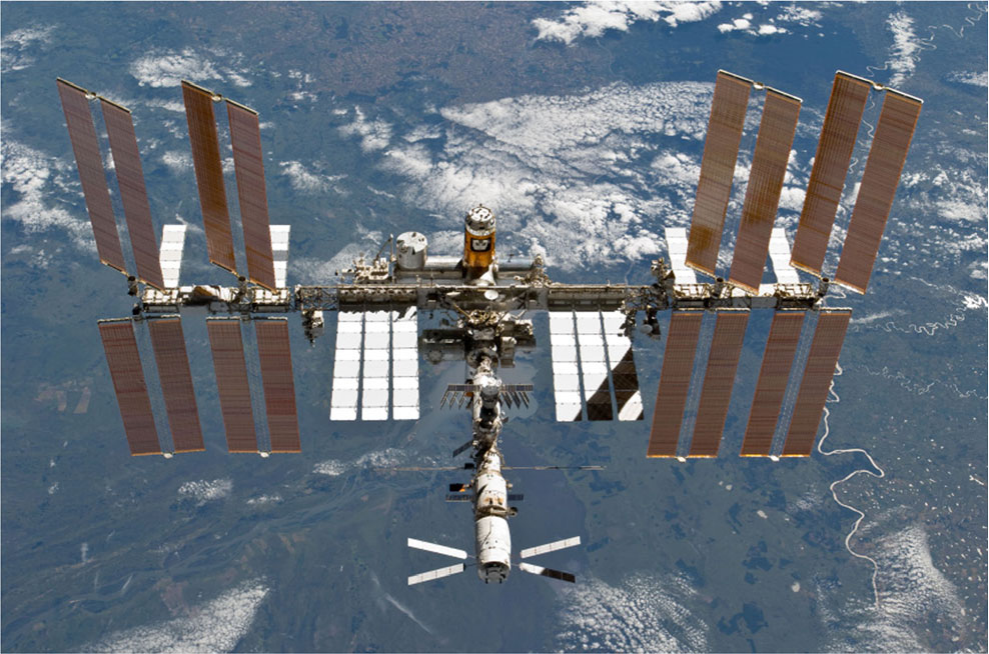
ISS in low earth orbit (courtesy of NASA)

With the Russian module Zarya, the first piece of the ISS was launched in 1998; the station was considered fully assembled in 2011 with the configuration shown in Fig. 1. Currently, a crew of six astronauts lives and works on board the station in the habitable volume of 388 m^3^. The total mass of the ISS amounts to 420 t; the overall outer dimensions measure 51 m in-flight direction and 109 m for the truss which carries the solar panels and the communication antennas. The ISS orbits the Earth with an inclination of 52.65° therefore covering 95 % of the world’s populated landmass. The time for one orbit is 93 min leading to a relative ground velocity of 7.3 km/s. Communication to ground is performed via UHF- and VHF-band directly to Earth and via S-, K_u_-, K_a_-band over data relay satellites.

The utilisation of the ISS actually focusses on the performance of scientific experiments using the special conditions of reduced gravity and increased radiation exposure. Scientific research facilities for experiments from human physiology, biology, materials science and fluid physics are implemented in the various laboratories inside the station and are operated and maintained by the astronauts [3]. The research laboratory Columbus represents the European contribution to the ISS. Experiments on astronomy, particle physics and earth observation, which demand the undisturbed view onto the surface of the Earth or into deep space, are placed outside the ISS on balconies and external platforms. They are mounted by a robotic arm or by astronauts in an extra-vehicular activity (EVA) and are remotely controlled during operation.

The residual acceleration on board the ISS ranges from 10^−4^–10^−6^ g depending on the location and on the operational schedule of the station and of the astronauts. Major sources of gravitational disturbances are orbit manoeuvres, the drag of the residual atmosphere which is noticeably present even in this altitude, and the physical activity of the crew members. Because of these numbers, it is more appropriate (and scientifically correct) to use the term *microgravity* instead of weightlessness when describing the environmental condition.

After the retirement of the Space Shuttle the ISS is supplied with consumables (including food, water, fuel, and equipment) by manned (Sojus) and unmanned (Progress) flights of Russian carriers, Japanese cargo vehicles, and private US enterprises (Space-X, Orbital).

For the upcoming years, the leading space agencies also plan to use the ISS for technology demonstration purposes and preparation of the envisaged exploration missions.

### Operation of the ISS

Hundreds of sensors are constantly monitoring environmental parameters like temperature, pressure, humidity, and concentrations of various gases (oxygen, carbon dioxide, ammonia, and volatile organic compounds (VOCs)) on board the ISS. These parameters are safety-relevant for crew health and fire detection; the related sensors must be highly reliable, robust and must possess a long life time. High-precision measurements of these parameters made available by deployment of innovative technologies are certainly of second priority in this context. These reasons favour the implementation of conventional sensor technologies rather than sophisticated measuring devices featuring state-of-the-art technologies for achieving high-precision data. To emphasize the importance of sensor reliability, it has to be noted that a single failure of one of the safety-relevant sensors—measuring the ammonia concentration in the station’s atmosphere—led to sealing-off the bulkhead of a single ISS laboratory compartment in January 2015 temporarily; in the worst case, such incident could lead to an evacuation of the station. It has to be kept in mind that due to its nature, the ISS does not represent a standard laboratory where the experimenter can open a window and get out in case of an accident.

Hence, an anomaly in the environmental conditions of the station has to be detected fast, reliably, and locally in order to initiate and execute corresponding countermeasures for protecting the health of the astronauts. If many similar sensors are distributed over the station, an irregularity can be detected and characterised quite early and also locally. To achieve this, the different sensors have to be connected to establish a sensor network. The related wiring, connectors, brackets and other mechanical parts to integrate the harness would sum up to a significant mass increase. However, the substitution of wired sensors by wireless sensors offers the opportunity to save mass and also integration effort for future applications.

This was the motivation for the German space agency, DLR, to foster the development of such a sensor network, the WiSe-Net (*Wireless Sensor Network*) on board the ISS.

### WiSe-Net

Wireless communication can be established via different technologies: narrow-band radio-frequency (RF) communication like WLAN (IEEE 802.11 [4]) or Bluetooth (IEEE 802.15.1 [5]) is used nearly everywhere today. However, these kinds of narrow-band communication are sensitive to interference and have some electromagnetic compatibility (EMC) issues. Therefore, ultra-wideband (UWB) RF communication (IEEE 802.15.4 [6]) has recently gained attention. Another technology insensitive to interference and EMC is optical communication in the visible or infrared range.

Wireless sensor networks not only have to communicate without wires but need to be supplied autonomously with electrical energy as well. To supply this energy, batteries can be used, but are used only when necessary on the ISS, due to the associated flammability and explosion risks. However, energy harvesting methods to convert available non-electrical energy are under investigation. There are three main energy sources available in space: (1) mechanical energy converted by piezo elements, (2) electromagnetic energy converted by appropriate circuits or by solar cells (optical range) and (3) thermal energy converted by thermoelectric devices.

#### Application on ISS

When looking at a possible use of a wireless sensor network on the ISS, a maximum range of 50 m × 60 m has to be considered, presuming the application is used within the pressurized modules. Though, considering a minimised number of base stations, the network should support a multi-hop network topology allowing forwarding data from sensor nodes not having direct contact to a base station. Such networks are supported by systems based on the IEEE 802.15.4 [6] standard. It operates on one of three possible unlicensed frequency bands, but only the band at 2.4 GHz is allowed worldwide and therefore preferred for application on ISS. On the other hand, Bluetooth and WLAN are operating in the same frequency range. Interferences with mobile devices (e.g., tablet PCs or laptops) may occur and disturb the operation of a wireless-sensor network. This becomes even more important, because the requirements for electromagnetic compatibility, which are applicable, are limiting the allowed emitting power significantly. This fits perfectly concerning the power demand for the sensor nodes, but makes it even worse with respect to the stability of the communication.

As an alternative an infrared (IR)-based communication system could be used. Concerning the mandatory regulations with respect to the safety of the station and especially for the crew on-board the ISS, such systems have clear advantages. IR seems furthermore attractive, because there is little equipment using this technology. On the other hand, the configuration of laboratory equipment, tools, and stored items on the ISS is permanently changed and hence the operational environment is not stable. If the astronauts are moving through the station, an IR-based communication is thus expected to be disturbed more frequently in comparison with RF-based systems. A detailed trade-off between several systems, taking into account availability of components and the maturity of the technology revealed that a RF-based system is the best option.

#### In-orbit demonstration of RF technology

In preparation of the mission of Alexander Gerst, which took place in 2014, the European Space Agency (ESA) and the German national agency DLR initiated a technology demonstration for wireless sensing technology on the ISS [7].

This was regarded as a chance to demonstrate the technology readiness for use in space applications and furthermore in the real ISS environment. Four sensor nodes located in the Columbus module consist only of commercial off-the-shelf components (COTS) which have been modified for use in space. Each of the nodes, with a volume of about 2 cm^3^, was equipped with environmental sensors for air humidity, air pressure, temperature, two-axis accelerometer and ambient light. The latter was selected to obtain a clear picture of the actual situation on-board. A further major objective was to operate the system without using any batteries, by using microelectromechanical (MEMS) devices in combination with low-power communication.

**Fig. 2:**
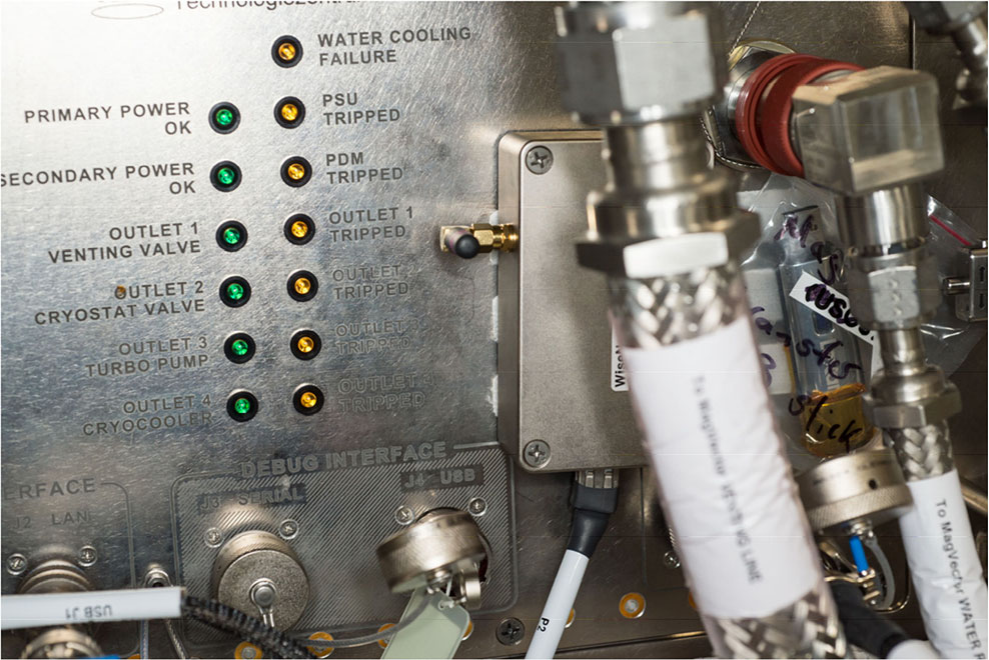
WiSe-Net base station (courtesy of NASA)

The base station of the network, as shown in Fig. 2, was placed at the front panel of an experiment insert. Furthermore, NASA required the limitation of the power to 1 mW.

**Fig. 3:**
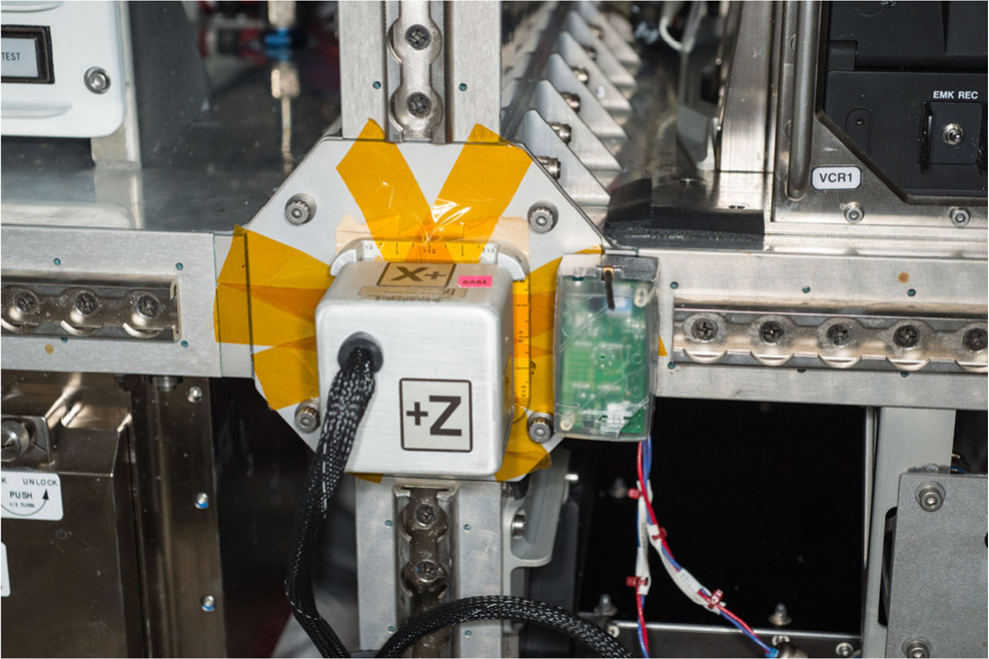
One WiSe-Net Sensor Node with transparent housing (courtesy of NASA)

The selection of the locations for the sensor nodes was determined with the aim at having different grades of influence on operational stability and different illumination conditions for energy conversion. Figure 3 is depicting one of the nodes, which represents a rather good location.

WiSe-Net was operated on board ISS in the period from December 2014 until March 2015. This technology demonstration was very successful since the controlled parameter exhibited a high stability with deviations of only a few percent. The evaluation of the data showed, that even though having a difficult environment, the RF communication was extremely stable with nearly no packet loss. The ability of the system to automatically establish a multi-hop network was not necessary. Each of the nodes had a direct link to the base station. Concerning energy provision it was shown that the use of ambient light as a PV power source was sufficient to avoid using batteries for the hardware used.

#### In-orbit demonstration of IR technology

In parallel to the preparation of the second run of the RF-based system, an infrared-based wireless sensor network is under development, that will be tested inside the ISS using the hardware infrastructure already used for the first WiSe-Net campaign as far as possible, to reduce the development effort to the necessary minimum.

The same set of sensors will be used for the wireless sensor nodes and they will be placed in the same positions as the sensors of the first WiSe-Net campaign to be able to compare the results with the already acquired data from the RF-based system.

To summarise, the first WiSe-Net campaign has shown the function of COTS based narrow-band RF wireless sensor network inside the ISS and has opened further possibilities to evaluate wireless sensor network technologies in space. The next steps will be an extension of the original WiSe-Net and an implementation of a comparable optical (IR) sensor network. As the wireless sensor network technology has been shown to be usable in the ISS environment, different applications are envisaged for the future, including gas hazard monitoring and Body Area Networks (BAN) for health parameter surveillance of astronauts, especially during extra-vehicular activities (EVAs).

## Research under reduced gravity

**Fig. 4:**
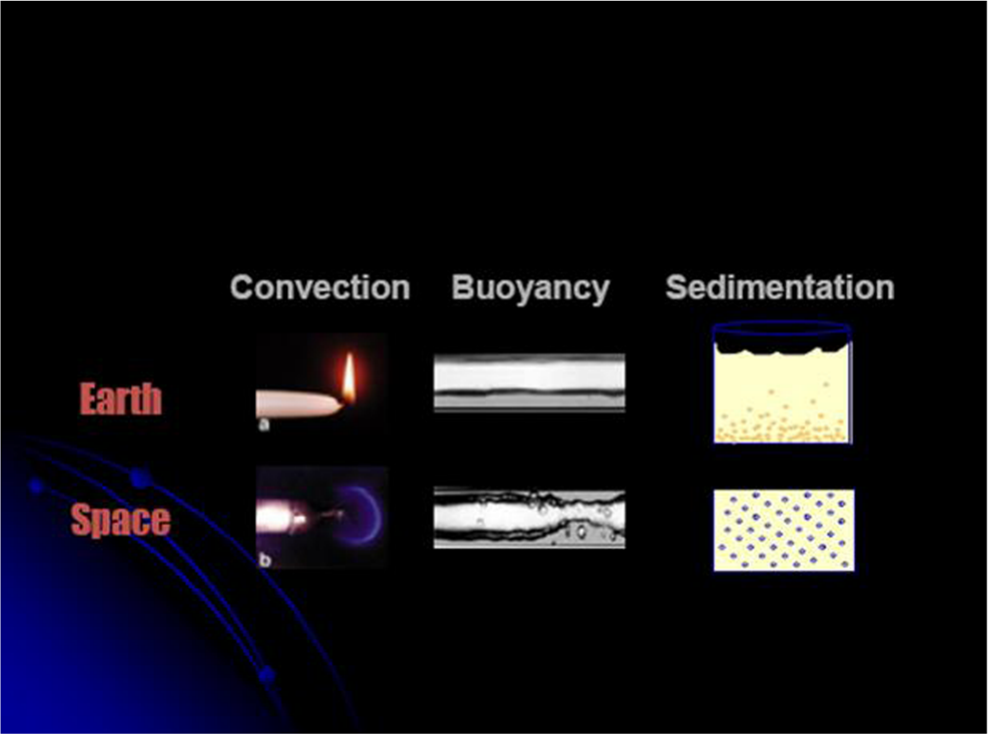
Effect of microgravity for a candle flame indicating the missing convection (*left*), a gas/liquid interface exhibiting the formation of bubbles under reduced gravity (*middle*), and the effect of sedimentation of solid particles in an emulsion (*right*) (courtesy of Georg Mason University, School of Public Policy)

The physical behaviour of matter under reduced gravity depends significantly on the phase condition: solid matter indeed freely floats under reduced gravity conditions, but without changing the macroscopic and microscopic properties of the body. The reason for this lies in the fact that the electromagnetic interaction which mainly determines features like the electronic structure, the related physical properties, and the chemical binding forces, is by orders of magnitudes larger than gravitational forces. However, for fluidic systems like fluids or gases a microgravity environment changes the macroscopic shape and interfaces and also the heat and mass transfer. In general, these phenomena facilitate processing without buoyancy-driven convection, hydrostatic pressure, and sedimentation. Ideally, the absence of gravity-driven buoyancy convection features a purely diffusive transport; the absence of sedimentation leads to homogeneous particle distribution in mixtures. Figure 4 illustrates the effect of microgravity on three examples.

Concerning materials science these effects are mainly relevant for experiments aimed at crystallisation of bulk materials from the fluid state (melt or vapour phase) and on the determination of thermo-physical parameters in the undercooled regime [8]. Crystal growth is interesting from a materials and biological standpoint. Protein crystallisation in microgravity allows the formation of perfect proteins, and is valuable in studying the efficacy of target protein binding (cell function, pharmaceuticals). Series production of materials on a large scale on the ISS is not, and will likely never be, a practical endeavour.

For fluid physics experiments, small capillary forces, which are independent of gravity, become dominant in addition to the absence of buoyancy. Hence, interface phenomena (capillary effects, drops and bubbles, behaviour of immiscible fluids), multi-phase flows (boiling and condensation, flow regimes and transitions) can be studied without the disturbing effect of gravitational forces. Capillarity is thereby caused by surface tension in fluid interfaces [9].

The mentioned effects are even more pronounced for combustion processes, but on the other side often overlaid by strong thermodynamic effects making a complete understanding of a complex combustion process rather difficult.

The situation is rather different for all biological creatures; these consist mainly of liquid water, leading to a significantly altered metabolism, signal proprioception, and signal transduction if exposed to microgravity. Various physiological processes and the distribution and motion of body fluids are affected by the microgravitational environment [10]. Long-term exposure of humans could result in muscle atrophy, osteoporosis, or fluid shift [11, 12]. Spaceflight has been shown to impact virulence, reduce the immune system, and impact the vestibular system. The growth of plants and the distribution of nutrients are also affected by different gravity conditions and the repair mechanisms of radiation damaged cells as well.

All experiments which have been performed so far in space were driven by scientific objectives—even if industrial, terrestrial applications were backing the execution in the dedicated environment of reduced gravity.

A more direct benefit of processing under reduced gravity can be found for cold atoms. Even if they have a very minute mass, an atomic beam experiences gravity on Earth and tend to follow a bomb trajectory. In a microgravity environment the beam particles freely move linearly without any acceleration. This can be used, for example, to extend the length of a detecting cavity in atomic clock [13].

For deep space investigations aiming at measuring electromagnetic radiation or massive particles, a detector located in Low Earth Orbit (LEO) is highly advantageous compared to a ground-based device since the Earth’s atmosphere reduces the counting rates for massive particles by several orders of magnitude for high-energy particles and absorbs most of the electromagnetic radiation.

## Categorisation of sensor systems

Before describing dedicated sensors and sensor systems in actual ISS experiments, a more generic view is given in this section in order to classify the different sensors and sensor systems which are deployed in scientific research facilities on board ISS.

**Fig. 5:**
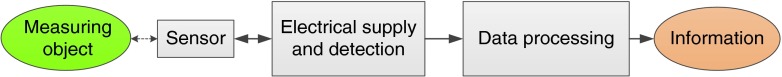
Schematic sketch of measurement process with a single sensor

In a classical reflection, a sensor represents a device which is in contact with the medium to be probed. The nature of this contact could be a direct physical one, a visual one, or could be established by a transfer medium. The sensor interacts with the medium in some way and gives the measured information to the outside. In ancient times, the reaction of the sensor on the measured object was mostly mechanical and normally the measurement information could be read on a scale. For example, lengths were measured by rulers with a pre-defined length scale printed on it; temperatures were measured by observing the thermal expansion of a liquid in a capillary, which is equipped with a calibrated temperature scale. Later, as the range and accuracy of the sensors have been extended, the sensors became more sophisticated and the mechanical reaction was replaced in many applications by an electrical interaction between object and sensor. Now, temperatures were measured by a change in the electrical resistivity and a length expansion by the piezo-electric effect. Hence, the information was comprised in an electrical signal, in most cases an electrical voltage. Electrical information then enables recording and electronic processing the measurement data and their implementation into large and dedicated control cycles. Figure 5 illustrates schematically the structure of such simple measurement process with a single sensor.

**Fig. 6:**
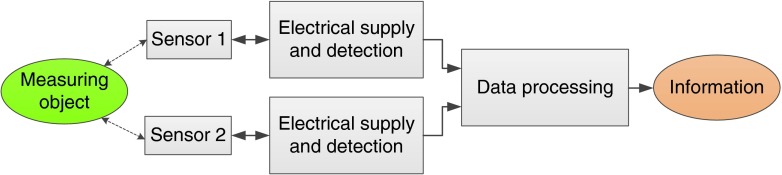
Schematic sketch of measurement process with a multiple sensors

In real applications, often two or more sensors are deployed in order to measure either the same quantity with a certain redundancy or a local distribution of this quantity extended over a certain volume. If the object to be measured is not homogeneous, for example, an atmosphere containing different gases, different sensors could be deployed each for detecting a distinct gas species. This multiple-sensor array scheme is depicted in Fig. 6.

**Fig. 7:**
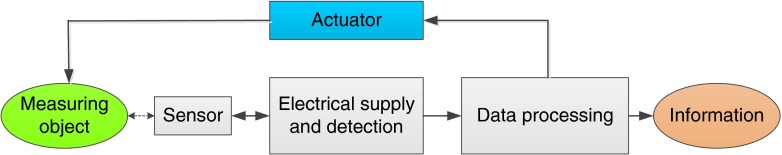
Schematic sketch of a simple control loop which takes the information gained from the measurement process as input for adjusting the actuator parameters accordingly

The measurement of a physical quantity denotes a necessary prerequisite for a closed loop control application. In this case, the sensor reading serves as input for a control system which controls the setting of the respective actuator that modifies the probe accordingly. For example, to keep a sample temperature in a well-defined range the control algorithm has to adjust the heater power settings according to the measured deviation of the sample temperature from the set-point value. For an optimum performance of the closed control loop the time constants of sensors, actuators, and the probe itself must be in the same order of magnitude to provide optimum conditions for a fast and accurate control of the respective quantity. This extended scheme is illustrated in Fig. 7.

**Fig. 8:**
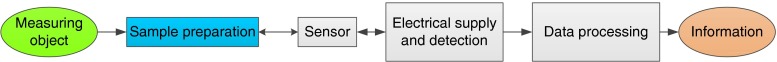
Conceptual layout of a measurement process taking a dedicated sample preparation into account

Measurements in life sciences are normally not directly accessible for the quantification. Here, a dedicated, multi-step sample preparation is often required as indicated in Fig. 8. Typical examples are: Sample taking from the subject of interest, mechanical treatment (ultrasound application for cell disruption to get access to the inside material), physical treatment (bring the sample into solution, temperature control or cycling (e.g. for polymerase chain reaction, PCR)), or chemical conditioning (well-defined chemical reaction to get a colorimetric reaction, which can be measured by a spectrophotometer, to preserve the actual sample condition for later analysis or the selective staining or labelling of the sample sites of interest, e.g. for life imaging, immune-assays etc.).

These macro-pre-processing steps are accompanied by exact volumetric addition of reagents and frequent washing steps, with low sample losses under controlled temperatures.

Similar to research in terrestrial facilities, research on board ISS makes use of sensor technologies in various ways. The sensor systems implemented in ISS research facilities and which are described in more detail below directly reflect the different types as described in the preceding section.

## Sensor systems for selected scientific payloads

In the course of this chapter, eight selected experiments for physical and life sciences on board the ISS will be introduced and described. Special emphasis is laid on the sensor systems and the related technology. First scientific results which have been gained with these sensor systems are reported. The selection of experiments was performed following two major objectives: (1) to give examples for all sensor categories as introduced in Chap. 4, (2) to cover a broad spectrum of scientific applications.

### Electromagnetic levitator

The research facility Electromagnetic Levitator (EML) serves for the measurement of thermo-physical properties of technologically relevant metals and metal alloys in the undercooled liquid state and for investigating solidification processes emerging from this state. The main advantage for performing these types of experiments under reduced gravity lies in the fact that this specific environment enables a free levitation of the spherically shaped molten sample without any contact to sample containment.

To achieve this, the samples with a diameter in the range of 6–8 mm are exposed to a superposition of two electromagnetic fields—a quadrupole and a dipole field. The quadrupole field forces the sample to move to the field minimum which is located in the centre of the coil system whereas the dipole field heats the sample by induction. The sample is indeed freely levitating in the reduced gravity of the ISS, but the quadrupole field is still needed to counteract residual forces arising from the movement of the astronauts, the residual atmospheric drag or attitude control manoeuvres of the ISS. This setup allows avoiding any containment of the sample which may lead to unwanted chemical reactions at high temperatures. Furthermore, the measurement of surface tension and viscosity can be conducted without any external disturbances. EML represents a nearly ideal environment for these measurements that could hardly be provided at terrestrial laboratories [14].

The EML facility has been launched in 2014 and is in operation on board the ISS since early 2015. Two additional diagnostic systems are currently being developed for this facility to extend the scientific research potential: The first system measures and controls the partial pressure of oxygen in the process atmosphere down to the sub-ppm range, and the second one—the Sample Couple Electronics (SCE)—determines the electrical conductivity of the levitated samples by using a slight change in the RF processing data.

#### Oxygen control system

The measurement of material properties at elevated temperatures requires often precise knowledge of the oxygen partial pressure (pO_2_) since surface oxidation and dissolving of oxygen in the sample material may impact the properties significantly. Depending on the considered substance, its oxidation may occur even at extremely low pO_2_ such as 10^−19^ Pa. Especially the measurement of viscosity and surface tension is rather sensitive to the contamination of the sample surface by small concentrations of oxygen. This was the motivation for developing the Oxygen Control System (OCS) for the EML facility. The OCS consists thereby of two major components, a potentiometric pO_2_ sensor and an oxygen titration pump.

Measurement of pO_2_ concentration at elevated temperatures using e.g. optical, photo-acoustic, resistive, or amperometric methods can hardly provide a sensitivity of better than 10^−1^ Pa. Therefore, electrochemical systems based on solid-state electrolytes seem to be the most suitable approach. They allow measurements of the pO_2_ in the range from 10^7^–10^−19^ Pa and exhibit very short response times.

The oxygen titration pump is based on an yttrium-stabilized zirconia (YSZ) operated at a temperature sufficiently high to enable the transport of O^2−^ through an electrolyte, but low enough to suppress the electronic conduction.

Therefore, both devices, the potentiometric sensor and the oxygen ion pump, rely on the potentiometric transport of oxygen ion through an electrolyte: The potentiometric sensor measures the Nernst potential directly, whereas oxygen is actively pumped through the electrolyte by applying a current. For this reason, the selected electrolyte can be used for both functions.

**Fig. 9:**
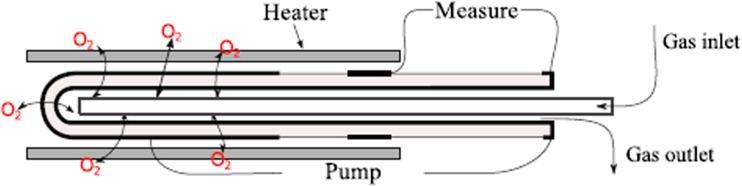
Conceptual sketch of the oxygen ion pump (courtesy of Technical University of Clausthal, Germany)

The potentiometric pO_2_ sensor consists of a one-side closed, 8 % Y-doped ZrO_2_ tube with platinum electrodes at its surfaces. The carrier gas is delivered through the inlet into the active area where the applied current controls the oxygen flow as shown in Fig. 9. Further downstream, the reference electrode measures the Nernst potential from which the partial pressure can be determined. The tube of the oxygen ion pump looks quite similar, but it is covered with platinum paste at three regions, i.e. the large pumping area, the reference electrode and the counter electrode. Due to the large distance between the pumping and reference electrode, no impact of the pumping current on the Nernst potential is observed.

Both the oxygen ion pump and the sensor are operated at a temperature in the range of 600–700 °C with a stability of ±0.1 °C. This relatively low temperature ensures sufficiently high oxygen ion mobility and long-term stability of the system as required for the electromagnetic levitation experiments. The pumping current *I* of up to 40 mA is adjusted by a pulse width modulation control based on a PID (proportional–integral–derivative) control algorithm. Thereby, the polarity of the current can be changed to allow bi-directional control of the pO_2_ pressure.

In contrast to conventional mass flow controllers, the oxygen ion pump can control oxygen flow with a resolution of at least 10^−10^ m^3^/s. In combination with a potentiometric sensor, it enables precise transport of oxygen into and out from the process chamber and thus allows precise control of the pO_2_ within the range of 10^−19^−10^4^ Pa [15].

Because of a strong nonlinearity of the pO_2_ with respect to the current *I*, classical PID algorithm cannot be used. The necessary modifications include a dynamic adjustment of PID parameters at different oxygen partial pressures. Further, the system features a cascade PID controller, which enables pO_2_ adjustment at two different potentiometric sensors.

A stand-alone system for oxygen partial pressure measurement and control has been developed and tested in the laboratory. This device served as technology precursor for the unit to be deployed in EML. In order to measure the oxygen partial pressures at two different positions within the system, an additional potentiometric sensor was built.

**Fig. 10:**
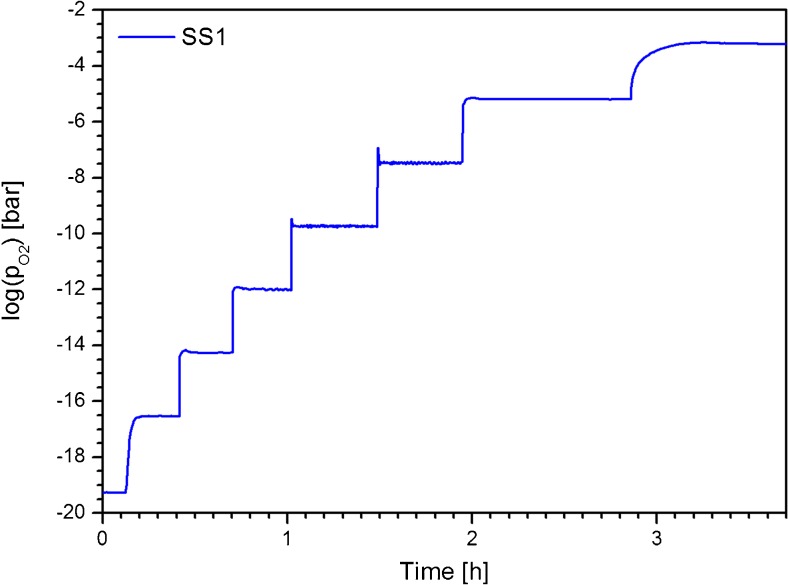
Control of the partial pressure of oxygen. The pO_2_ values which have been measured in the test campaign with an additional potentiometric sensor (named *SS1*) are indicated by the *blue line* in this figure

The test chamber was filled and operated with Argon gas of 99.999 % purity (pO_2_ = 1.6 × 10^1^ Pa). The gas flow rate was set to 2.5 × 10^−4^ m^3^/s. The response time of the system was determined predominantly by the total volume of the experimental chamber. With this setup, a control of the pO_2_ within 10^2^ to 10^−15^ Pa range with an uncertainty of Δlog(pO_2_/Pa) < 0.02 could be verified as shown in Fig. 10.

**Fig. 11:**
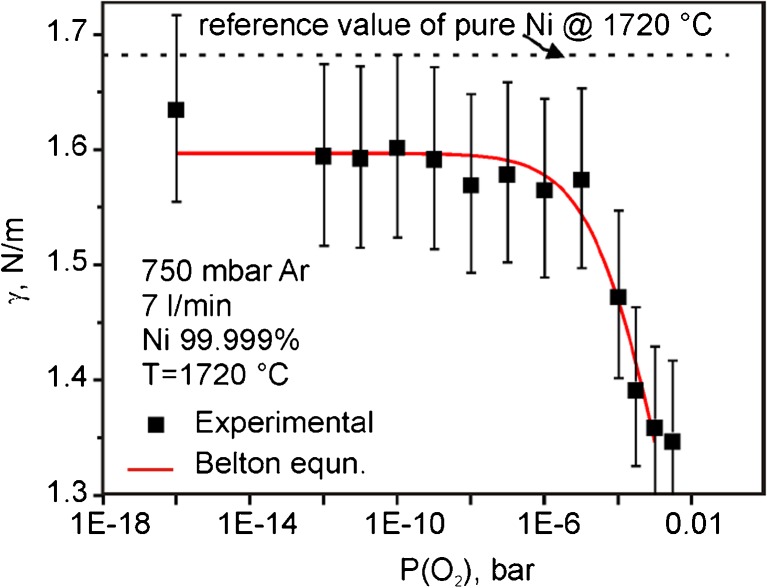
Surface tension of Ni as measured with the OCS in a levitation facility. The *solid red line* represents the Belton equation, a good agreement is evident [16]

In a second step, the system has been successfully tested in the vicinity of high power sources of electromagnetic fields, like, e.g. power supply for electromagnetic levitation facility. The surface tension of molten Ni determined as a function of oxygen partial pressure shows a good agreement with the Belton Equation (see Fig. 11). It confirms the correct operation of OCS setup as well as the impact of pO_2_ on the metal’s surface properties at elevated temperatures.

#### Electrical conductivity sensors

**Fig. 12:**
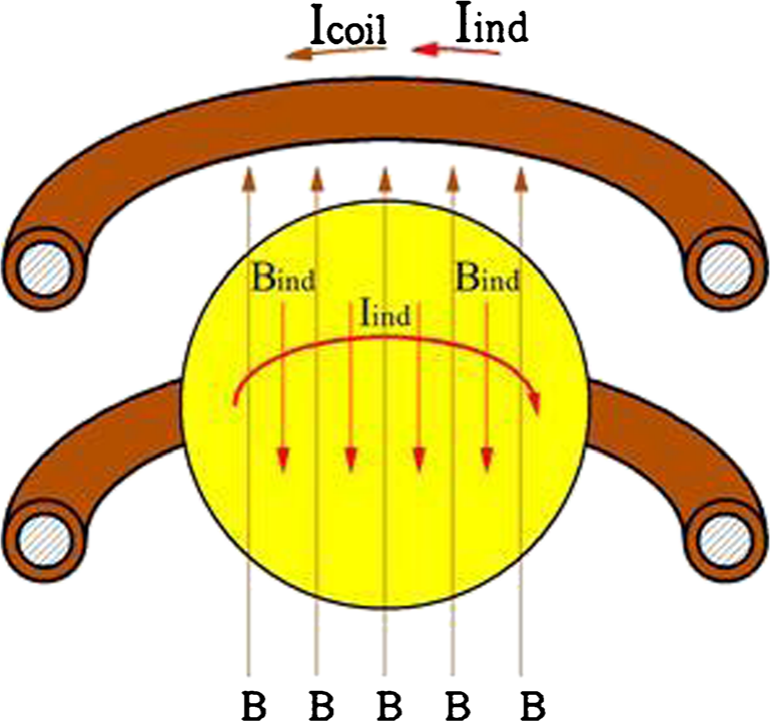
Schematic sketch of the RF field induced by the coil current (courtesy of DLR)

The electrical conductivity of the levitated sample represents one of the interesting thermo-physical parameters which can be determined with the EML facility. Conventional measurement methods rely mostly on contacting the sample with thin electrical wires which could impose a contact resistance or a chemical reaction at the contact point, both falsifying the measurement of the electrical conductivity. EML offers the possibility to melt and undercool metallic samples in a levitated state. The electrical conductivity can be measured by the inductive feedback of the sample to the RF system of the coil: The electromagnetic dipole field generates eddy currents in the sample depending on the electrical conductivity. These eddy currents themselves generate an additional induction field which results in an additional current in the coil (see Fig. 12). This applied technique is called sample coupling electronics (SCE).

Using a dedicated electronics, the data for coil current and voltage as modified by the inductive feedback can be measured with a relative precision of 10^−4^. Therefore, the electrical conductivity can be determined with a precision of better than 1 % [17].

**Fig. 13:**
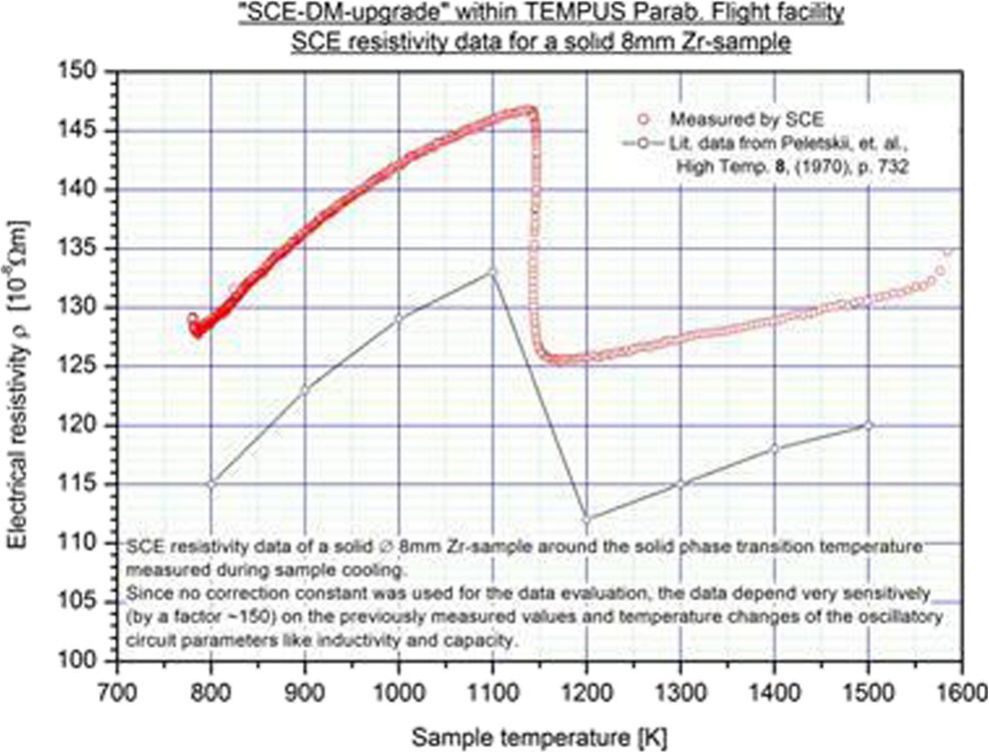
Electrical conductivity versus temperature for Zr below the melting point as measured with a laboratory version of the SCE sensor system. The drop slightly at 1150 K represents a phase transition in the solid Zr; the *solid line* illustrates literature data (courtesy of DLR)

The SCE system is going to be launched in late 2016 to the ISS and to be implemented into the EML facility. Functional test with the ground model have been performed and compared to literature data. The electrical conductivity for solid Zr versus temperature is plotted in Fig. 13. The steep decrease at around 1150 K is caused by a phase change in solid Zr. The comparison of both data sets shows a clear correlation of the slope with a certain off-set in the absolute values. This could be related to systematic differences in the test setup.

### High-precision temperature control of a multi-zone furnace

Materials science research in space covers investigations in crystal growth, direct solidification, diffusion, and the measurement of thermo-physical properties [18]. The general objectives for performing such experiments in space are:

- To understand fundamental physical mechanism and processes
- To validate numerical models
- To provide benchmark data for industrially important materials

One key aspect influencing the microstructure in solidification experiments is the shape of the solid–liquid interface in the solidifying material. At low growth rates, the interface is planar like for semi-conductors. Industrially relevant metallic materials crystallise at much higher growth rates. As the rate of growth increases, the interface develops a corrugated texture until three dimensional cells form in the solid. Even higher growth rates cause the formation of dendrites. The development of these different interface shapes and the transition from one shape to another is controlled by the morphological stability of the interface. Gravity represents one of the most influencing factors to this stability. In particular, buoyancy-driven convection can influence the stability and, thus, the shape of the solidifying interface. Hence, processing under reduced gravity imposes therefore well-defined conditions for crystallisation [19].

In the recent decades, the subsequently listed examples of most striking results have been obtained in microgravity experiments:

- Study of morphological stability in immiscible systems [20]
- Processing of eutectics [21]
- Melt interface stability studied by Seebeck coefficient variation [22]
- Dispersed systems [23]
- Dendritic growth [24]

The temperature stability at the solid-liquid interface represents a further significant parameter for the control of the morphological stability of the interface. The modular experimental facility Materials Science Laboratory (MSL) [25] is successfully being operated since 2009. Presently, it allows performing investigations on the above-mentioned issues. MSL can support different heater inserts which generate the final thermal profile at the inserted sample. For example, the Low Gradient Furnace (LGF) possesses seven individually controllable temperature zones which allow adjusting the Bridgman-type temperature gradient in the range of 2–40 K/mm.

To establish high-precision temperature stability on a short term, three major aspects have to be considered:

- Sensors with an intrinsic high resolution and a small time constant
- Actuators whose time constant is correlated to the respective sensor element
- A dedicated control algorithm

The application of sheathed thermocouples represents the standard technique for temperature measurement and control in furnaces. The achieved stability amounts to about 0.1 °C [26]. Within the MSL facility type C (W-Re) thermocouples, insulated by HfO with a sheath diameter of 0.7 mm are applied. In order to prevent a degradation of the Ta sheath due to a reaction with the graphite diffusor, they were inserted into protection tubes out of Ta and these are implemented into axial borings in the graphite diffusors. The single heaters consist of encapsulated graphite heaters where a meandered graphite layer (50 μm thick) is deposited onto a pBN (pyrolytic boron nitride) substrate. The heating layer is covered by a second pBN CVD (chemical vapour deposition) layer to protect it against mechanical, electrical, and chemical injuries.

In order to provide a fast and effective temperature control, a dedicated control algorithm has to be established which reflects the thermal coupling between neighbouring zones in addition to the implementation of sensors and actuators with similar, low time constants [27]. The developed high-precision temperature control system is based on a holistic view of the furnace, reflecting sensors, heaters, software, and control algorithm. Since the individual zones are not adiabatically separated from each other, an initial temperature difference between the zones causes thermal fluxes which affect the temperatures of the zones again. Hence, there exists a strong thermal coupling between zones and the furnace is to be regarded as a multiple-input multiple-output (MIMO) plant. In order to develop an optimised feedback controller, the thermal properties of each zone as well as the couplings have to be known exactly.

In a first step, a theoretical furnace model based on conduction and radiation between the zones is established, and then the unknown parameters are experimentally determined by process identification. This means measuring the dynamic temperature changes as response to well-defined power signals (pseudo random binary signal). These semi-empirical data represent the input for the parameter estimation algorithm which finds optimised model parameters. The obtained model is basis for feedback controller synthesis [28]. The final controller consists of a separate PID algorithm for each zone with pre-filter, pilot control, decoupling and anti-reset-wind-up.

**Fig. 14:**
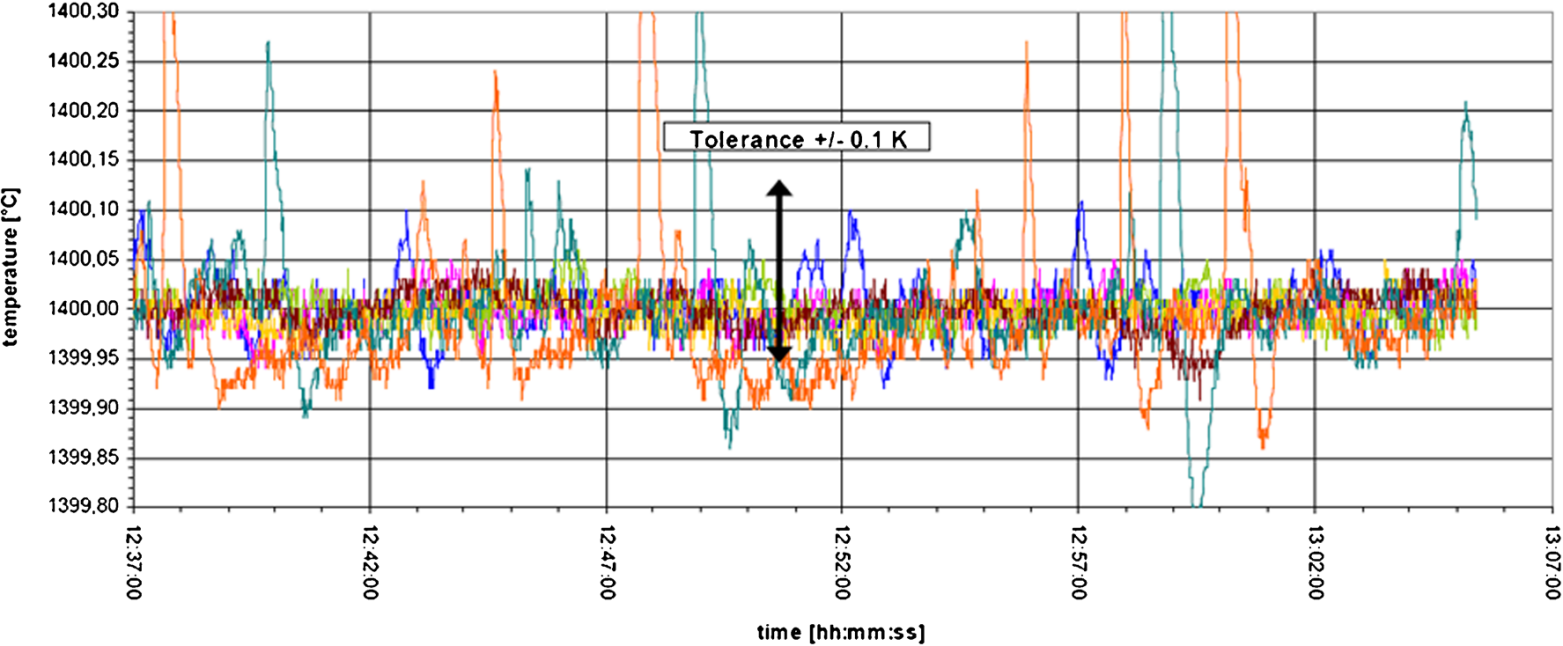
Course of temperature for all seven zones of the multi-zone solidification furnace LGF on ISS (courtesy of ESA)

This combination out of an adapted hardware and a sophisticated control algorithm has enabled to control the temperature on a mid-term with unrivalled precision. In Fig. 14, the course of temperature of all seven heated zones is illustrated over a period of 30 min. A relative temperature stability of *T* = 0.05 °C at *T* = 1400 °C could be achieved.

### IMMUNOLAB

**Fig. 15:**
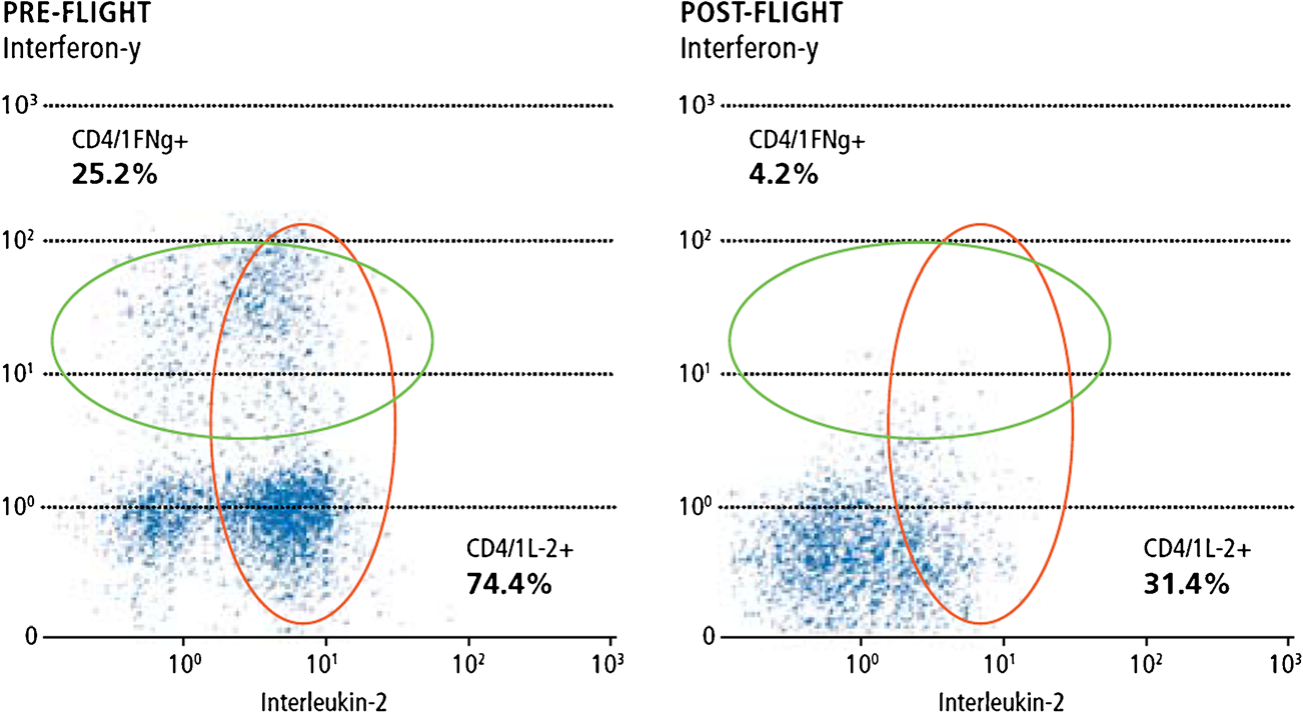
Plotted number on the appearance of two different cytokines before and after a space flight. The post-flight data clearly indicate that the number of cytokines has been significantly reduced [29]

First immunologic investigations on astronauts which were exposed to microgravity for a longer duration showed a significant reduction of the immune system. One aspect was that the T-cells were no longer capable to produce a sufficiently large number of cytokines which represent the messenger substances of the immune system (see Fig. 15). The reason for that has not been clarified up to today [29]. This phenomenological observation has motivated the German Space Agency DLR to initiate a dedicated research programme on the human immune system when exposed to the conditions of reduced gravity. This IMMUNOLAB project shall also give valuable information on the preparation of manned long-term missions to Mars [30].

**Fig. 16:**
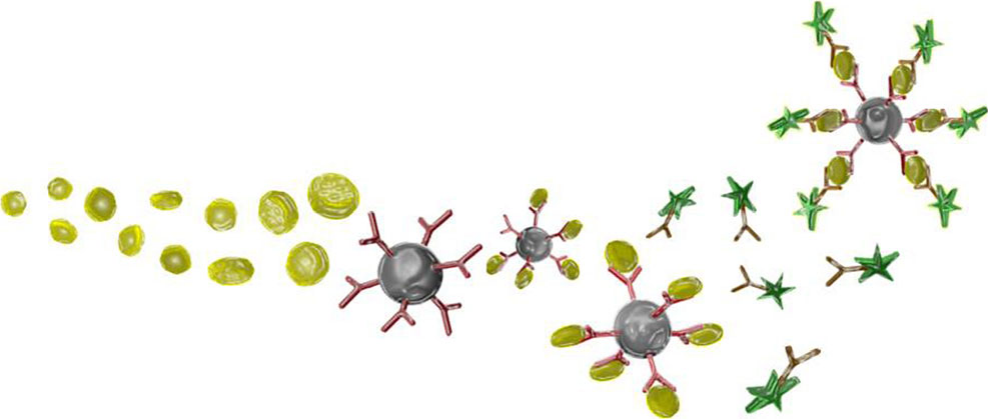
Selective labelling and *immobilisation* of a cytokine (*left*) by chemical docking of anti-bodies (*y-structure*) and fluorophore substances (*dark green*) for optical detection/quantification via fluorescence microscopy (top right)

The health status and the subjective stress level of a human can be analysed by measuring different blood parameters or characteristic features of the blood cells. The concentration and composition of the different cytokines in the blood are key parameters of the performance of the immune system [31–33]. Hence, the in-flight quantification of cytokines is the major task of IMMUNOLAB. Cytokines themselves are much too small for direct optical observation; therefore they dock to specific anti-bodies and to fluorophore substances in order to make them visible for optical microscopy (see Fig. 16). This approach allows the quantification down to below 1 pg/ml for samples of 1–100 μl.

**Fig. 17:**
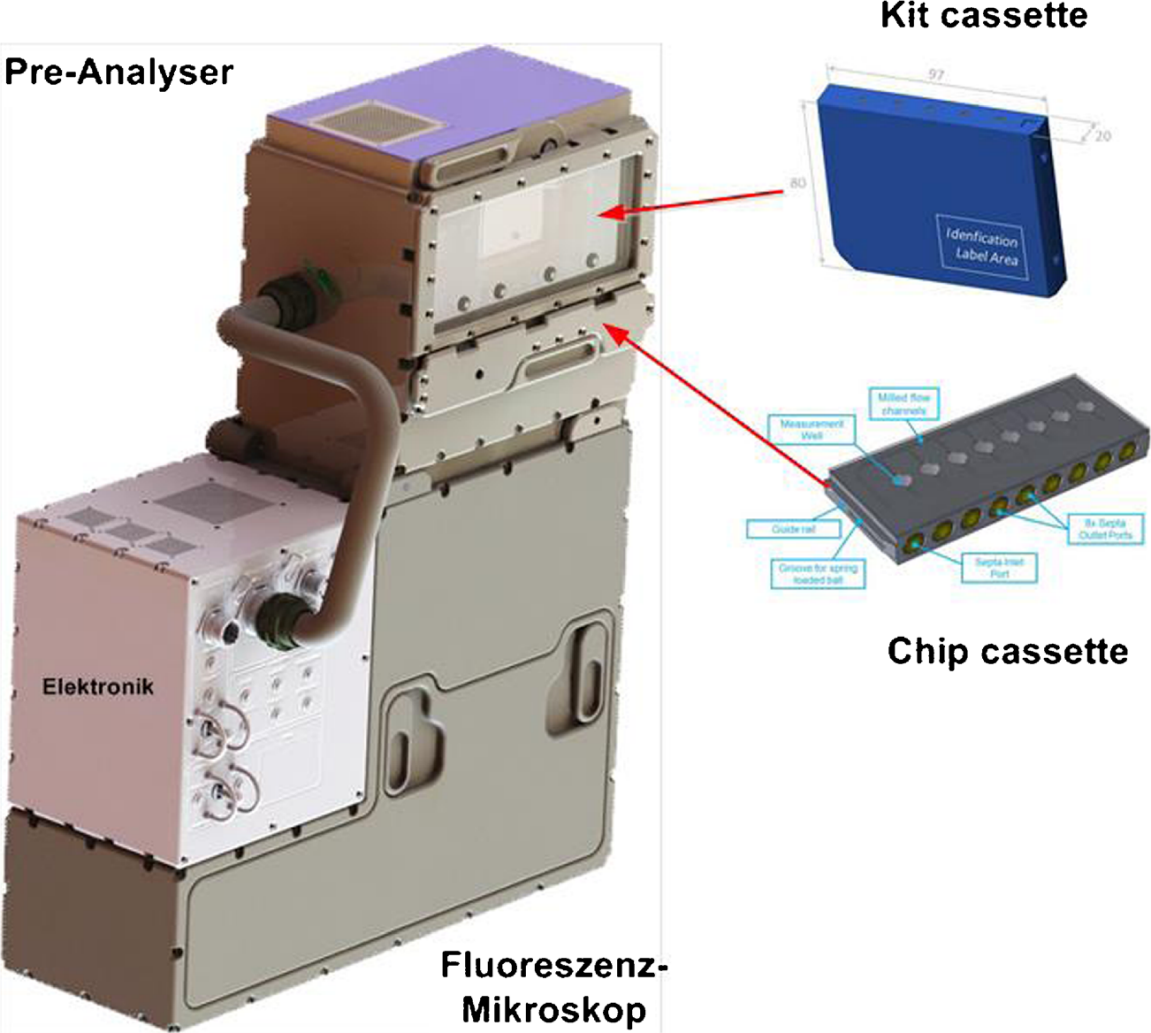
A conceptual sketch of the IMMUNOLAB facility used for blood analysis on the ISS

Since it is very complex to establish an immunologic laboratory on ISS having the same high quality standards as terrestrial laboratories, the complete process starting from taking a blood sample until the final evaluation of the fluorescence measurements has to be automated as far as possible. The main emphasis of the development of IMMUNOLAB has been laid to this dedicated aspect. The facility provides the necessary infrastructure for the analysis of human samples like blood, urine, saliva and blood cells. It combines sample preparation using quality-controlled commercial analysis kits and detection via fluorescence microscopy as analyser in one system as illustrated in Fig. 17.

On-board the ISS, the astronauts will take the relevant samples (blood, urine or saliva). The samples will be filled into a chip cassette for further automatic pre-processing to perform an immune-assay. The quantitative analysis is being performed in the analyser by measuring the intensity of the parameter-specific fluorescence signal. In order to utilise a broad spectrum of commercial analysis kits, the application of common fluorophores like fluorescein isothiocyanate (FITC), R-phycoerythrin cyanine, PE and Cy5 is supported by IMMUNOLAB.

IMMUNOLAB facilitates a reproducible in-flight, on-board analysis of the samples with high precision and an easy operation. This feature enables the investigation of short- and long-term effects on the immune system of the crew in the range of six orders of magnitude, starting at a few pg/ml. Hence, IMMUNOLAB represents a first step for an automatic assessment of the health status of astronauts during space missions and the gained data could contribute to enhance the basic knowledge on the functionality and performance of the human immune system as well.

The IMMUNOLAB is the first technological corner stone for the support of long-term exploration missions, up to the flight to Mars. These missions require a high autonomy in on-board diagnostics of the crew without direct support from Earth. The on-board diagnostic is a basic technology to support also other applications as monitoring of the life support system, control of the environment monitoring of the microbial contamination of a spacecraft. The technology is used for the test of suitable cleaning procedures on a small scale, and the verification of the cleaning efficiency after large-scale applications.

### E-Nose

The growth of microorganisms can represent an important issue concerning crew health especially when envisaging long-term space missions. A second, more technical issue was the detection of microbial corrosion. First hints of practical impact of the biological contamination have been gained at the Russian Mir station. Over the 15 years of operation of the Mir Space Station, 108 bacterial and 126 fungal species were identified [34]. A preferred location for colonization was the humidity condensate that accumulated behind the facility panels and racks. The analyses were performed post-flight on Earth by means of conventional methods after collecting samples from the contaminated surfaces or filters and sending them down. It was concluded that a permanently manned space station can provide a proper milieu for spreading or accumulating on various materials. Depending on the substrate, the microorganisms may deteriorate or even damage the outer structure, cabin interior, and equipment by biological corrosion. The environmental conditions on the ISS are quite similar to those of the Mir station, meaning a well-controlled temperature around 20 °C, high humidity, and appropriate sources of organic material could foster the uncontrolled growth of microorganisms [35]. Hence, it seems essential to have a reliable detection system for biological contaminants in order to take countermeasures already in an early stage. It was also evident that the analysis has to be performed on board, during the flight with a portable sensor system which can be brought to the contaminated locations and not vice versa.

This was the motivation for DLR to start the development of the E-Nose, a portable sensor system which is able to detect in situ bio-contamination at the relevant locations and surfaces on board the ISS in regular intervals [36].

**Fig. 18:**
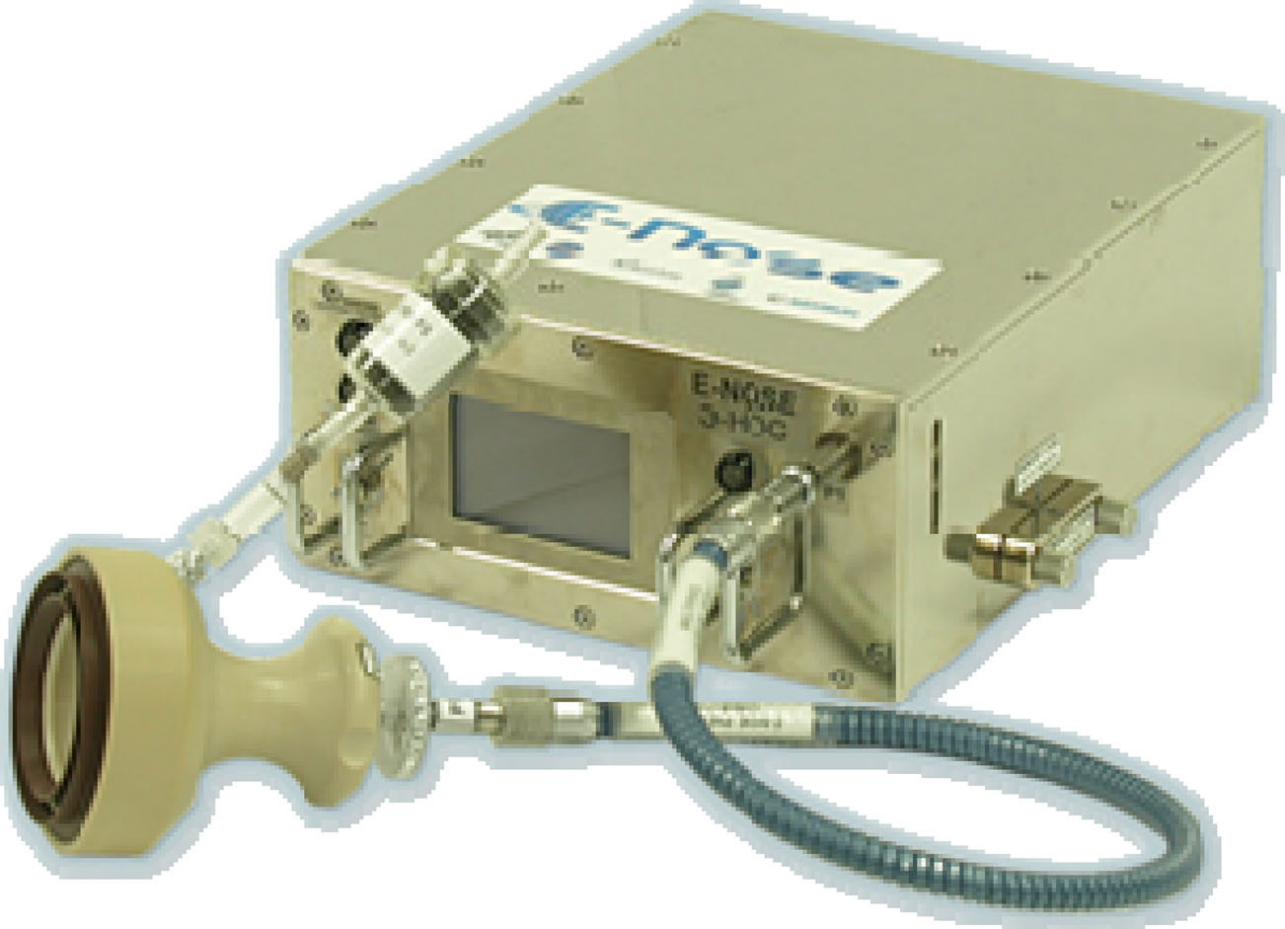
E-Nose as developed for utilisation on board the ISS (courtesy of DLR [36])

The E-Nose sensor system consists of 10 metal oxide sensors having specific sensitivities against different chemical gases. It is based on a commercial sensor system (*Portable Electronic Nose* from AIRSENSE Analytics GmbH, Schwerin, Germany) using the signal patterns of the sensor array. For utilisation on board ISS, this device has been modified with respect to housing, mechanical structure, electrical supply, and data handling; but the original metal oxide sensors have not been altered (see Fig. 18). These are sensitive versus hydrogen, sulphur, methane, alcohols, chlorine, aromatic and aliphatic gases and are mounted closed to each other forming an array—the “E-Nose”—with a diameter of about 40 mm. The different sensors measure a characteristic odour signature of the probed sample making use of the reducing or oxidizing properties of the biomolecules which are emitted from the samples (so-called MVOCs: microbial volatile organic compounds). These MVOCs are formed by the metabolism of the biological cultures and are highly specific for each kind. From the specific excitation of the E-Nose with respect to sensor type and signal strength, the E-Nose can be trained to determine a characteristic olfactory finger print for a dedicated sample, e.g. a spot with bacteria or microorganisms. The positive identification requires a detailed teaching of the analyser S/W. It considers the change of the signal pattern due to the different stages of the life cycle of the microorganisms [37]. It should be mentioned that the E-Nose does not measure directly the MVOCs. It requires a number of dedicated steps including the collection of the sample gas by a conditioned air stream.

**Fig. 19:**
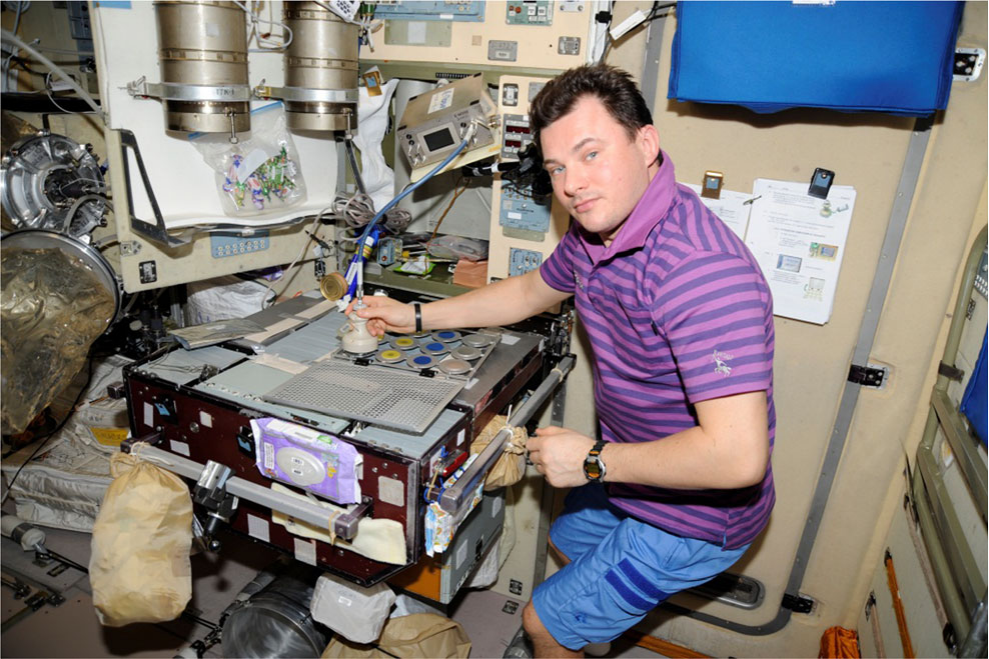
Astronaut working with the E-Nose in the Russian “Zvezda” module (courtesy of NASA)

First experiments in space have been recently performed in the Russian “Zvesda” module of the ISS. The aim of these investigations was a technology demonstration of the developed E-Nose under realistic environmental conditions on the ISS for several months performed in three campaigns. In detail, three different locations (working table, sleeping cabin and toilet lid) were probed with the E-Nose in each campaign (see Fig. 19). After the end of the last campaign, all locations were swabbed with Q-tips, and the samples swab probes as well as data from the E-Nose were brought back to Earth for analysis. On Earth, the in-space measurements were compared with an odour database containing four organisms, but a consensus odour could not be identified. Microbiological results also could not provide clues to the smell that was actually measured. After a literature research, the yeast *Rhodotorula mucilaginosa* was identified as the most likely candidate for the unknown odour. Further investigations showed that the smell of *R. mucilaginosa* matches very well with the data obtained inside the ISS. Finally DNA from *R. mucilaginosa* was proven at Q-tip 2 taken from the sleeping cabin of the cosmonaut and which confirms the assumption that the yeast *R. mucilaginosa* was actually measured in space by the E-Nose [37]. These first results clearly showed the feasibility of detecting bio-contamination in space with the E-Nose and elucidated the benefit of having a straightforward, liquid and label-free detection method on board ISS. It is therefore conceivable that the E-Nose will be part of the diagnostic equipment for the detection of microorganisms for long-term space missions. Currently, an upgraded version of the E-Nose is being sent to the ISS again for executing a second large measurement period. It is also planned to use the E-Nose for breath gas analysis for the monitoring of the astronauts. First studies on parabolic flight campaigns have revealed successful results.

### FIPEX

In low earth orbit (LEO), the residual O_2_ molecules absorb the incident sun energy, are split up into atomic oxygen, and accumulate in the upper atmosphere. The interaction of the solar radiation with the Earth’s atmosphere plays hereby a dominant role. The concentration of atomic oxygen depends on the sun cycles, the position, and on the Earth’s magnetic field. Atomic oxygen can lead to corrosion effects of exposed surfaces like optics and solar arrays of spacecrafts which are orbiting Earth in these altitudes. This phenomenon is well known as *Shuttle-glow*.

Reliable and systematic data on the temporal and local distribution versus the parameters mentioned above are missing. This was the motivation for DLR to fund the development of the FIPEX experiment (*Flux-(Phi)-Probe-Experiment*) for measuring the concentration of atomic and molecular oxygen on an external platform of the ISS at the Technical University of Dresden [38]. Finally, FIPEX has been space-qualified, launched, and operated on ISS and measured scientific data during February 2008 and October 2009.

**Fig. 20:**
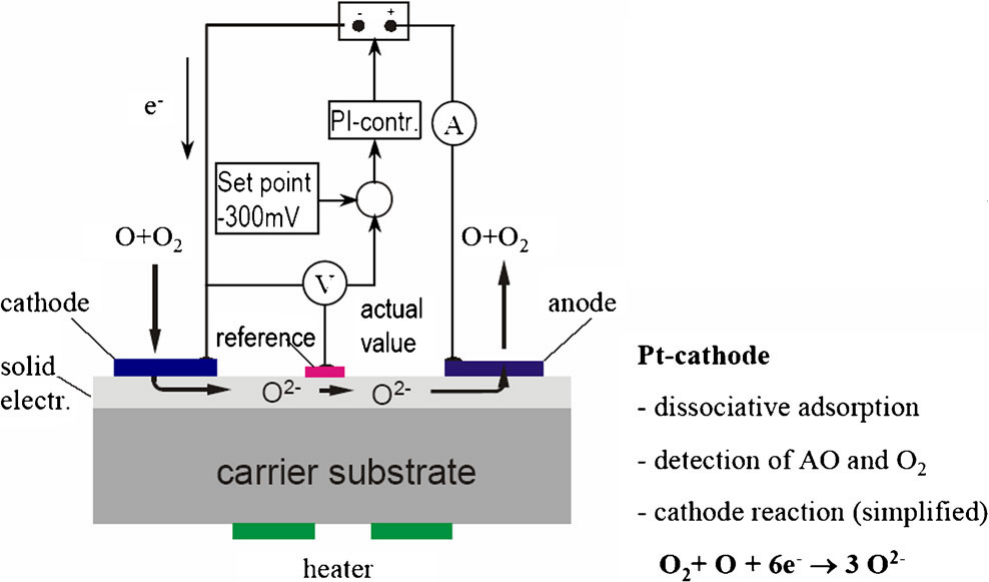
Schematic sketch of the FIPEX sensor package [38]

The FIPEX experiment consists of two sensor units which contain, in total, 12 amperometric oxygen sensors as schematically shown in Fig. 20. These kinds of sensors can distinguish between atomic and molecular oxygen. They convert the flux of oxygen atoms through an electrochemical cell with a diffusion barrier on top into an electrical current. The cell is made of yttrium-doped zirconia. The FIPEX sensors combine thereby the amperometric measuring principle with a flat, miniaturised design resulting in short response times. The electrolyte is silk-screen printed and itself part of the diffusion barrier. The required porosity can be adjusted by additional layers on top of the electrolyte. Atomic oxygen was measured via non-dissociative absorption, whereas the dissociative absorption senses the sum of both atomic and molecular oxygen. These sensor packages were capable to measure the oxygen partial pressure down to 10^−8^ Pa and work at a temperature of 650 °C.

**Fig. 21:**
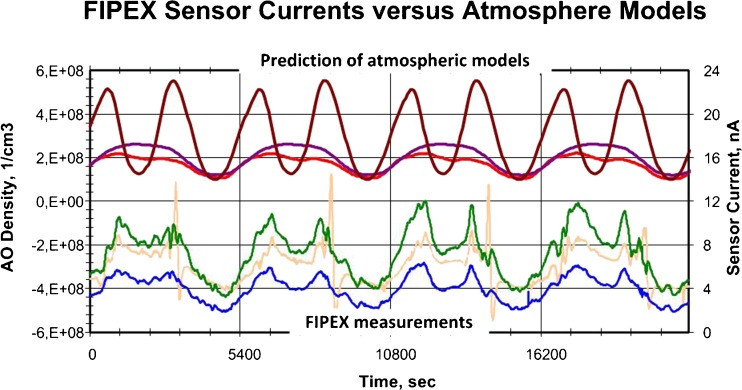
Comparison of FIPEX data (*lower three lines*, corresponding to right *y*-axis) with predictions of different atmospheric models (*upper three lines*, corresponding to left *y*-axis) [38]

Since the FIPEX experiment was mounted on the ISS which orbits the Earth roughly 16 times during 1 day, the local distribution of atomic and molecular oxygen could sampled over the mission period of 572 days and the temporal evolution at a fixed position could be determined. This allowed testing different atmospheric models with respect to concentration, distribution, and temporal evolution for the first time. Figure 21 gives an example of the comparison between the FIPEX data and the predictions of different theoretical models on Earth’s atmosphere.

### Cosmic radiation detector AMS-02

Planet Earth is being hit by radiation from deep space of different kinds: electromagnetic radiation ranging from radio waves until high-energetic gamma-rays and also particle radiation reaching from light particles like electrons up to heavy ions and anti-matter. The Earth’s atmosphere absorbs most of the electromagnetic radiation except the visible and radar wavelengths and reduces the massive particle radiation at least by a factor of 10^6^. Hence, it is advisable to locate radiation detectors outside the Earth’s atmosphere in order to measure radiation from deep space with sufficient statistics. For measuring electromagnetic radiation, there is a long tradition of implementing dedicated detectors on board of satellites like Hubble Space Telescope, Herschel, Planck, ROSAT, etc. [39].

For measuring massive particles, only a few attempts have been made in the recent years to measure the mass and energy distribution of high-energy particles from deep space [40], [41]. Especially, the measurement of anti-matter particles has become a subject in the physics community since it could give hints on the existence and origin of dark matter [42]. Because anti-matter is rather rare compared to ordinary matter, the detection is quite difficult and arduous. Furthermore, the detection of anti-matter particles is overshadowed by a high flux of ordinary matter particles having the same charge. Hence, the detection of positrons, the anti-matter equivalents of the ordinary electrons, stands always in competition with the detection of ordinary protons emerging from deep space with a flux which is several orders of magnitude higher. This fact represented the limitation concerning resolution for all hitherto experiments on the determination of the massive particle radiation from deep space performed on board a space craft orbiting Earth in high altitudes [41].

For these reasons, a number of 500 researchers from 16 countries formed a scientist community aiming at measuring the massive particle radiation from deep space (protons, anti-protons, electrons, positrons, gamma-radiation, anti-Helium, Be-, C- and Fe-nuclei) with unprecedented accuracy and resolution. Under the leadership of Prof. Sam Ting from MIT, the team developed and qualified the Alpha Magnetic Spectrometer (AMS-02) for operation on board the ISS [43, 44]. AMS-02 consists of six different sensor systems which are each time sensitive to a dedicated particle type:

- Silicon tracker
- Transition Radiation Detector (TRD)
- Time-of-Flight counter (TOF)
- Anti-coincidence counter (ACC)
- Cerenkov Detector (RICH)
- Electromagnetic calorimeter (ECAL)

**Fig. 22:**
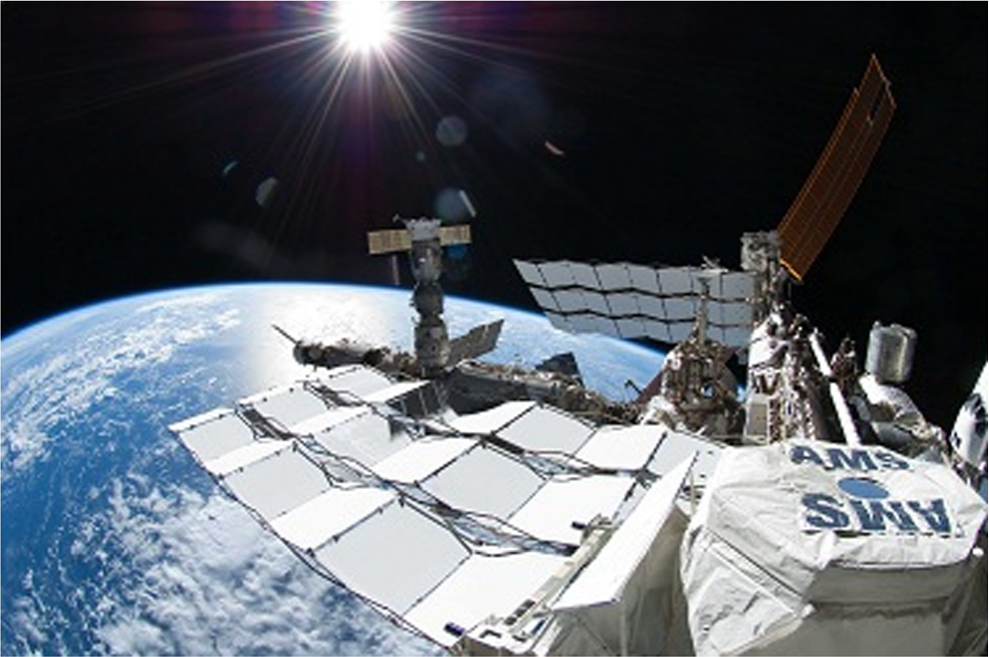
AMS-02 mounted on an external platform of the ISS (courtesy of NASA)

A large 0.1 T magnet acts a mass separator. AMS-02 weighs 8.5 t, has a volume of 64 m^3^, and consumes about 2500 W electrical power. It has been launched in 2011 with the last shuttle flight to the ISS and envisages an operational time of 10 years, which started in 2013 (see Fig. 22). Each incident particle leaves a characteristic trace in the six detectors. By evaluating the signals of the different detectors the mass, charge, and energy of the incident particle can be distinguished and measured.

Researchers at RWTH in Aachen have developed the TRD instrument which represents one of the key elements for the detection of positrons. In a transition radiation detector, a high-energy charged particle traverses the boundary of two layers having different dielectric constants in a 20 mm thick fleece and creates characteristic electromagnetic radiation which is measured in an ensemble of gas detectors following the multi-wire chamber concept. These detectors consist of straw tube modules filled with Xe/CO_2_ (80 %/20 %) gas. The TRD consists of 20 layers of straw/fleece modules mounted in a conical octagon structure. The length of the 328 straw modules varies from bottom to top layer from 0.8 to 2.0 m. Each straw module consists of 16 straws. The straws have an inner diameter of 6 mm; the wall material is a multilayer aluminium-Kapton foil with a total thickness of 72 μm. Gold-plated, 30 μm thick tungsten wires, coaxially mounted and fixed in a polycarbonate end piece, are used as sense wires. The TRD for AMS-02 is able to discriminate one positron under 10^6^ protons [45].

The accurate measurement of positron spectra could give a clear indication for neutralino annihilation with rather precise determination of the neutralino’s mass [42].

**Fig. 23:**
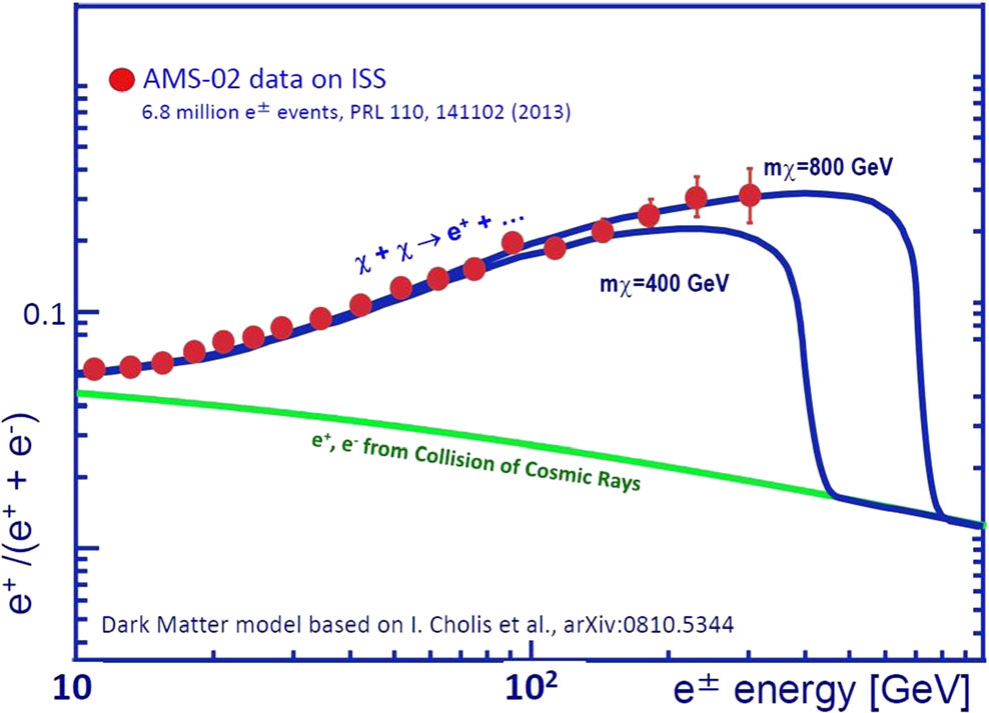
Measured positron excess compared to the prediction of theoretical models on dark matter assuming different masses for the neutralino particles [47]

The first measurements with the TRD yielded a positron excess compared to the prediction of the standard model of the universe (see green line in Fig. 23). The positron fraction which is defined as the ratio between the number of measured positrons and the sum of electrons and positrons decreases up to energies of 10 GeV as expected from the standard model, but from 10–250 GeV this ratio is steadily increasing. One potential explanation could be the annihilation of massive dark matter particles called *neutralinos* or χ-particles into positrons, gamma-rays, and anti-protons. In Fig. 23, the positron fraction recently measured by the AMS-02 instrument is drawn by the red dots. The two blue lines indicate theoretical predictions assuming different masses of the neutralinos [46, 47]. It is expected that the high accuracy of the instrument will further allow discriminating between the different theoretical predictions for the mass of these neutralinos in the upcoming years as indicated in Fig. 23.

### ACES

Accurate time measurement has become mandatory for exact navigation, verification of basic laws in fundamental physics, and for metrology purposes [48]. The technological progress in cold atom physics has enabled time measurement with accuracy in the 10^−16^ range (i.e. an error margin of 1 s within 300 million years) [49]. Cs fountain clocks represent today the time standard at all major national metrology institutes [13]. To operate a high-precision clock in LEO would bring the following advantages compared to an Earth-based clock:

- Synchronization of terrestrial clocks
- Precision tests of fundamental laws of physics
  - ○ Gravitational red-shift:
    As a direct consequence of Einstein’s Equivalence Principle (EEP), a radiation source in a gravitational field appears to an observer, which resides in a gravitational field with a different strength, shifted in frequency. The gravitational red-shift shall be measured by ACES with a relative uncertainty of about 3 × 10^−6^.
  - ○ Drift of the fine structure constant
    The atomic fine structure constant α, which determines the strength of the electromagnetic interaction in physics, is postulated as constant over time following the general relativity theory. In order to test models which predict a violation of this theory—a time variation of this and other fundamental constants, a high-precision clock like ACES shall be capable to measure a possible drift of α at an accuracy of around of δα/*t* < 10^−16^ per year [50].
  - ○ Anisotropy of the speed of light
    According to Einstein’s special relativity theory the speed of light *c* is isotropic, i.e. light is spreading with the same velocity in all directions. The ACES mission shall test the validity of this theorem at a level of *δc*/*c* < 10^−10^.
- Test of high-performance atomic clocks for space applications
- Verification of the feasibility for a high-performance time and frequency transfer

**Fig. 24:**
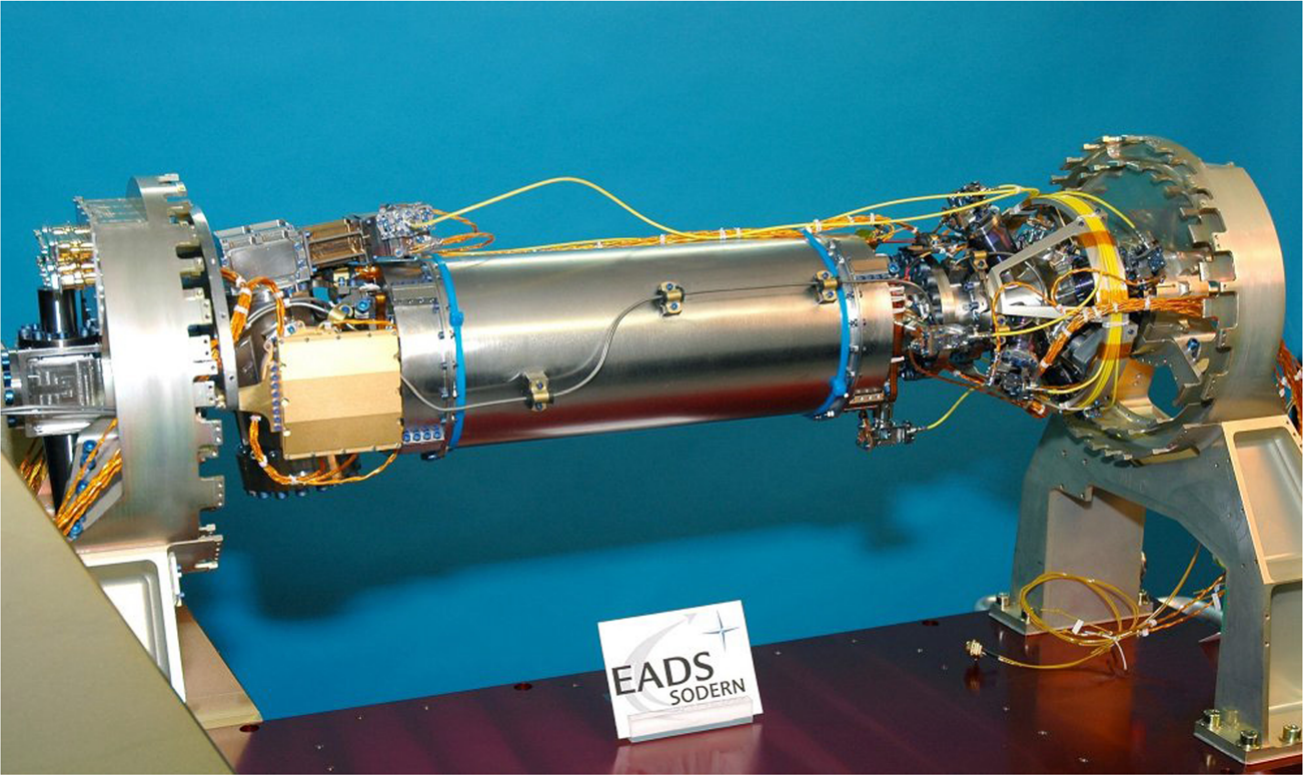
Cs atomic clock PHARAO before integration into ACES (courtesy of ESA)

Aiming to meet the stated objectives, the European Space Agency ESA has started the development of the *Atomic Clock Ensemble in Space (ACES)* in the late 1990s [51]. In order to improve the accuracy and stability of conventional Cs fountain clocks, ACES was designed as a combination of two high-precision atomic clocks, a Cs fountain clock PHARAO and a hydrogen maser SHM. PHARAO was developed by the French space agency CNES; it measures the hyperfine transition at 9.192631770 GHz (SI definition of the second) of Cs atoms which are cooled by laser beams to a temperature of 1 μK (see Fig. 24). The advantage of a Cs clock operating in space lies in the fact that the interrogation length with the microwave field is larger by a factor of 3 due to the straight movement of the atoms compared to the flight parabola as resulting under Earth’s gravity. PHARAO provides a clock signal with fractional frequency instability below 1 *×* 10^−13^ × *t*^−1/2^, where *t* represents the integration time in seconds and inaccuracy in the 1 *×* 10^−16^. The SHM operates at the hyperfine transition of atomic hydrogen at 1.420405751 GHz. The hydrogen gas is thereby confined in a glass bulb and the microwave frequency is tuned to the resonance. The fractional frequency instability for the maser varies with time; it amounts to 1.5 × 10^−13^ for 1 s interrogation time.

**Fig. 25:**
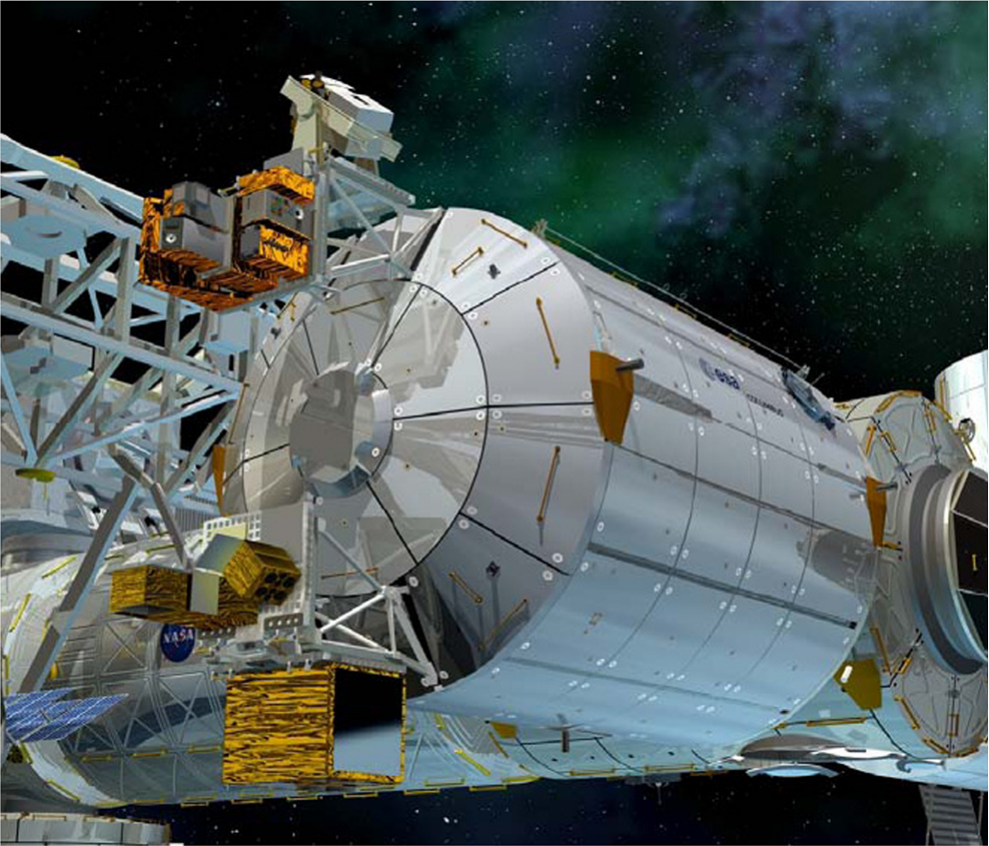
ACES payload (*large rectangular box on lower side*) mounted at an outer platform of the Columbus module of the ISS (courtesy of ESA)

ACES combines the good medium-term frequency stability of the SHM maser with the long-term stability and accuracy of a primary frequency standard based on cold atoms as delivered by PHARAO. The on-board clock-to-clock comparison and the distribution of the clock signal are ensured by the Frequency Comparison and Distribution Package (FCDP), which measures and optimises the performance of ACES. Finally, the FCDP distributes the 100 MHz clock signal to the microwave link (MWL) for transmission to several ground stations widely distributed over the globe. The envisaged clock-to-clock comparison in the 10^−17^ regime can be possible only if the link has an excellent long-term stability (up to 10 days of integration time). The ACES facility is planned to be launched in 2017 and to be mounted on an outer platform of the ISS as illustrated in Fig. 25.

## Critical review

The description of the various selected experiments and the applied sensor technology has revealed a rather multifarious picture. It became evident that even for the limited number of the eight described examples no common definition of the term *sensor* can be identified. The sensor element moreover must be regarded as one constituent of the complete measuring chain as elucidated by the sketches of the different scenarios in Chapter 4. The introduced ISS experiments can be thereby assigned to the different categories with respect to the characterisation of the sensor elements:

- Category 1—single sensor: SCE
  This example marks the only case where a single sensor measures directly a physical quantity of the sample in a standard way. But even this case offers already a unique feature: The sensor is identical with the respective actuator; the coil which produces the RF field for heating senses itself the change in electrical conductivity induced by the heating process.
- Category 2—multiple sensors: E-Nose, AMS-02, FIPEX, ACES, WiSe-Net
  In these instances, several sensors are deployed to probe the specimen under different aspects:
  - Location (WiSe-Net)
  - Gas species (FIPEX, E-Nose)
  - Charge, mass, and energy (AMS-02)
  - Time regimes (ACES)
  From the measured sensor signals, the originally interesting physical quantity is created by subsequent electronic data processing.
- Category 3—Sensor as substantial part of control loop: MSL, OCS
  Here, the sensing elements are serving as inputs for a dedicated control loop to regulate a distinct physical parameter.
  The achieved control stability for the LGF clearly manifests that not the performance of the sensor element provides the unrivalled short-term temperature stability, but moreover the complete measurement chain. The single sensor element, in this case a conventional type C thermocouple, is not a dedicated development aiming at achieving an improved performance by deploying a new measurement technology or manufacturing process of the element itself. The figure of merit lies in the appropriate combination of sensor, actuator, control algorithm, and furnace design; all these systems have to be matched to each other, the perfect interplay between these elements finally results in the optimum reaction of the control system to occurring disturbances and the obtained temperature stability of less than 0.1 °C at 1400 °C.
  A similar situation is obvious for the OCS: The premium control stability of the pO_2_ within the range of 10^−19^−10^4^ Pa can only be achieved by combining a potentiometric sensor with a titration oxygen pump.
- Category 4—Sensor systems with dedicated sample preparation: IMMUNOLAB
  As representative for numerous life science experiments, IMMUNOLAB can only develop its full capability in the measurement process if an appropriate sample preparation is applied before. The original detection system relies on conventional, imaging fluorescence microscopy. Improving the performance of this element only would not be sufficient to enhance the overall sensitivity and performance of the instrument. The preparation process of the sample before a fluorescence measurement is also essential for this goal. However, this aspect reflects the situation at a dedicated point in time; if envisaging a larger time period in order to study systematic changes features like reliability and repeatability become more and more important. Regarding the complete picture of the measurement process, a single aspect like the sensitivity of the sensing element alone gets definitely a lower priority. To succeed in reaching the original goal, an end-to-end thinking of the complete measurement process must be endorsed, starting at the critical analysis of the objectives, reaching over the establishment of a suited measurement concept, sample collection, and pre-processing method, and ending in the accurate realisation of the single elements in the measurement chain.
  The statements argued above are certainly valid for complex terrestrial measurement tasks as well. Distributed sensor networks have not been invented for space applications. A high-precision control of the short-term stability in a high-temperature furnace or of the pO_2_ at low concentrations could be installed in terrestrial laboratories and facilities in the same way. Automated blood analysis is being performed on Earth all day with high precision. High-energy particle detectors with a high sensitivity have been operated in large terrestrial laboratories for decades. The specific reasons for performing the introduced experiments in space are elucidated in the preceding chapters already. In this section, a closer look on the specific aspects which are additionally imposed to these measurement systems for an application in space shall be conducted.
  In a first step hereto, the boundary conditions related to space operation shall be explained and some examples for corresponding countermeasures introduced.
  The application in space requires for any technical system an extensive qualification and testing programme. This also holds for sensors and the related equipment. The key issues to be addressed are:
  - The system must survive the launch into space: During launch the payloads suffer severe mechanical loads, up to 10 g static acceleration and dynamic loads (root-mean-square acceleration) up to 7 g^2^ in the frequency range of 1–1000 Hz.
  - The system must withstand the radiation from deep space without any loss of performance:
    - ○ This depends on the final orbit, for example the radiation doses on board ISS are roughly 100 times higher than on Earth. This issue is particularly important for electronic systems of all kinds including electronic boards, computers, storage devices, cameras, and displays. By radiation hardening, some of the occurring failures can be prevented.
  - It shall be operational for the mission duration without any degradation, for ISS payloads this means 5 years, for planetary mission much longer. This requirement imposes a high reliability and robustness of the system which has to be verified in extensive tests prior to the implementation.
  - The sensor system should operate with a high degree of autonomy. Although the astronauts on board ISS could perform some action like inserting, exchanging, or enabling sensors, the crew time is a valuable resource and therefore the payloads should be operated as autonomous as possible. On unmanned carriers, a complete autonomy is required.
  - The system should work without the need of any maintenance operation. This issue is relevant for sensor systems which have to be re-filled or re-supplied with a necessary means of production. For example, the available amount of cooling liquid determines the operational life time of cooled sensors and often the duration of the mission (PLANCK, Herschel satellites).
  - Environmental conditions: Temperature and pressure inside the ISS are well-controlled. But for the experiments on outer platforms which are exposed to space environment temperature variations from −50 °C to 120 °C could occur.
  - In the manned, closed environment, ISS NASA imposes high safety regulations for any technical equipment: The payload must not endanger the health of astronauts, the integrity of the ISS and its equipment, and the ISS operations. Thus, special emphasis has to be laid on the utilisation of toxic chemicals, shatterable and flammable materials. Offgasing of materials has to be investigated and the electrical supply has to be checked versus safety regulations.
  Often, even science-economic reasons drive the development of payloads and innovative sensor systems as well, meaning that the investment in building and operating a research facility in space must be accompanied by a substantial gain in performance. This was the reason for driving the LGF control system in space to its technical limits and to improve the long-term accuracy of an atomic clock by adding a second one to form the ACES ensemble.

The development of compact sensor systems for life sciences like E-Nose or IMMUNOLAB, however, was imposed by a rather specific demand: to have a reliable, mainly automated measurement system which allows systematic in-flight, on-board diagnostics over a long time period and requiring minimal ground support. These achievements will create their spin-offs into terrestrial applications where similar demands are apparent—on autonomous facilities with strongly reduced links and exchange to the external world like on research stations at extreme environments (South Pole, deserts), submarines, oil platforms, caves, disaster support, etc.

To summarise, scientific research in space and especially on board the ISS fosters the development of robust, mostly autonomous, and reliable sensor systems. The single sensor element is thereby always embedded in the complete measurement chain, which is rather specific to the respective experiment. A holistic view of the measurement problem enables to deploy the optimum solution to the problem. Applying these technologies in space yields not only an optimum performance of the respective scientific experiments which is certainly the major objective of this undertaking, but offers also often an excellent opportunity for field test of the developed sensor technologies and methodologies under the harsh and extreme conditions on board ISS. Furthermore, the application in a hostile environment like space often pushes the innovation of systems with high performance and/or compact design and mode of operation. Hence, a large potential of spin-offs into terrestrial applications can be identified.

The Wise-Net sensor network represents a good example for the statements above: The successful test campaign on ISS has shown that it is feasible to operate a wireless sensor network under the extraordinary conditions of a manned space station with limited volume, disturbing RF noise all over the place, and operating astronauts. The next step is now the application of such a network in the Ariane rocket; the implementation of an RF- or IR-based network would save 100 kg weight on cables. The final development step could be the implementation of such networks in airplanes. The verification in space application is to be regarded as field test for such commercial, civil applications.

The dedicated development and qualification processes for space projects in general lead to a time span between the first ideas for an experiment until its actual execution in space of more than 5 years. As a consequence, the implemented technologies are often no longer state-of-the-art. On one hand, technical teething troubles of new technologies are avoided in this case, but on the other hand new development cannot be considered, and sometimes compatibility problems could arise.

Vice versa, space research can benefit from terrestrial sensor developments and progress by implementing new technologies and methods into the space projects. The space research facilities will always profit from innovations in the sensor elements and in the measurement methodology. The great challenge lies in the appropriate introduction into the respective measurement concept and detailed realisation.
